# Effect of Insoles with a Toe-Grip Bar on Toe Function and Standing Balance in Healthy Young Women: A Randomized Controlled Trial

**DOI:** 10.1155/2017/2941095

**Published:** 2017-11-14

**Authors:** Hideki Nakano, Shin Murata, Teppei Abiko, Masashi Sakamoto, Dai Matsuo, Michio Kawaguchi, Youji Sugo, Hiroaki Matsui

**Affiliations:** ^1^Faculty of Health Science, Kyoto Tachibana University, 34 Yamada-cho, Oyake, Yamashina-ku, Kyoto 607-8175, Japan; ^2^ASICS Trading Company Limited, 3-5-2 Yasakadai, Suma-ku, Kobe City, Hyogo 654-0161, Japan

## Abstract

**Objective:**

The aim of this randomized controlled study was to investigate the effects of insoles with a toe-grip bar on toe function and standing balance in healthy young women.

**Methods:**

Thirty female subjects were randomly assigned to an intervention group or a control group. The intervention group wore shoes with insoles with a toe-grip bar. The control group wore shoes with general insoles. Both groups wore the shoes for 4 weeks, 5 times per week, 9 hours per day. Toe-grip strength, toe flexibility, static balance (total trajectory length and envelope area of the center of pressure), and dynamic balance (functional reach test) were measured before and after the intervention.

**Results:**

Significant interactions were observed for toe-grip strength and toe flexibility (*F* = 12.53, *p* < 0.01; *F* = 5.84, *p* < 0.05, resp.), with significant improvement in the intervention group compared with that in the control group. Post hoc comparisons revealed that both groups showed significant improvement in toe-grip strength (*p* < 0.01 and *p* < 0.05, resp.), with higher benefits observed for the intervention group (*p* < 0.01). Conversely, no significant interaction was observed in the total trajectory length, envelope area, and functional reach test.

**Conclusions:**

This study suggests that insoles with a toe-grip bar contribute to improvements in toe-grip strength and toe flexibility in healthy young women.

## 1. Introduction

Falls are among the most common and serious problems in older adults [1]. More than one-third of individuals who are ≥65 years of age fall each year, and falls are recurrent in 50% individuals [2]. Motor functional declines like muscle weakness, gait deficit, and balance deficit are main risk factors of falls [1], especially toe flexor muscles associated with gait and balance ability in older adults [3, 4]. Misu et al. reported that decreased strength of the toe flexor correlated with slower walking speed, shorter periods of the single-limb support phase, and shorter stride length during fast-pace walking in community-dwelling older adults [3]. Furthermore, Menz et al. showed that the strength of the toe flexor muscles is a significant independent predictor of balance and functional ability in older adults [4]. Some studies have reported that toe flexor strength decreases with aging [5–10] and have shown that low toe flexor strength is an important risk factor for falls among older adults [11, 12]. We also reported that toe-grip strength (TGS) was significantly decreased in older adults compared with that in healthy individuals [13]. TGS muscle weakness is an important risk factor for falls because it is significantly decreased in older adults who have experienced falls compared with those who have not [14]. We have previously investigated the TGS training effect on fall prevention in older adults [15] and showed that training improved TGS and decreased the fall rate in the year after training. We also previously demonstrated that center of gravity sway in healthy young women was reduced when they wore shoes with insoles with a toe-grip bar compared with that when wearing shoes with general insoles or when standing barefoot [16].

Previous studies have reported that regular exercises such as progressive resistance training in older adults [17] and towel-gathering exercises in young adults [18] are effective for increasing toe flexor strength. However, the regular exercises are impeded by personal, environmental, and activity characteristics [19]. Therefore, toe-strength training, which is unimpeded by those characteristics, is needed. Insoles that can be used conveniently are an effective tool to solve the above problems. An advantage of the insole intervention is that individuals can perform it easily and regularly because it only requires walking using the insoles. Hence, the intervention is unlikely to be affected by personal, environmental, and activity factors. Previous studies have shown that insoles can improve standing balance in healthy individuals [20–22] and older adults [23–28]. Moreover, it was reported that insoles can alter the muscle activity and strength of lower limb [29–31]. From these studies, insoles with a toe-grip bar may be an effective tool to improve standing balance and muscle strength.

In previous studies, we showed that towel-gathering exercises increased TGS in young women [18] and that center of gravity sway in young women was reduced when they wore shoes with insoles with a toe-grip bar [16]. Thus, an intervention using insoles with a toe-grip bar may improve TGS and standing balance in young adults, and if so, it is plausible that older adults with muscle weakness and balance disorder may experience similar benefits. Hence, the present study was conducted with young adults as an initial study before considering implementation of such an intervention for older adults.

The aim of this randomized controlled study was to investigate the effects of insoles with a toe-grip bar on toe function and standing balance in healthy young women.

## 2. Methods

### 2.1. Ethics Statement

The study conformed to the Declaration of Helsinki, and the Ethical Committee for Research of Kyoto Tachibana University approved the protocol (approval number 16-1). All subjects gave their written informed consent and were free to withdraw from the study at any time. This study was registered in UMIN Clinical Trials Registry (UMIN000026697). The present study was conducted at Kyoto Tachibana University between April and September 2016.

### 2.2. Participants

Thirty healthy women [mean age ± standard deviation (SD): 19.97 ± 0.71 years; mean height: 157.68 ± 4.60 cm; mean bodyweight: 51.36 ± 4.62 kg] participated in this study. All participants were recruited from Kyoto Tachibana University. Only women were recruited because the fall rate and risk in women are significantly higher than in men [32]. Participants were excluded if they had a chronic (orthopedic, neurological, or psychiatric) disease that might influence the results.

### 2.3. Procedures

**Figure 1:**
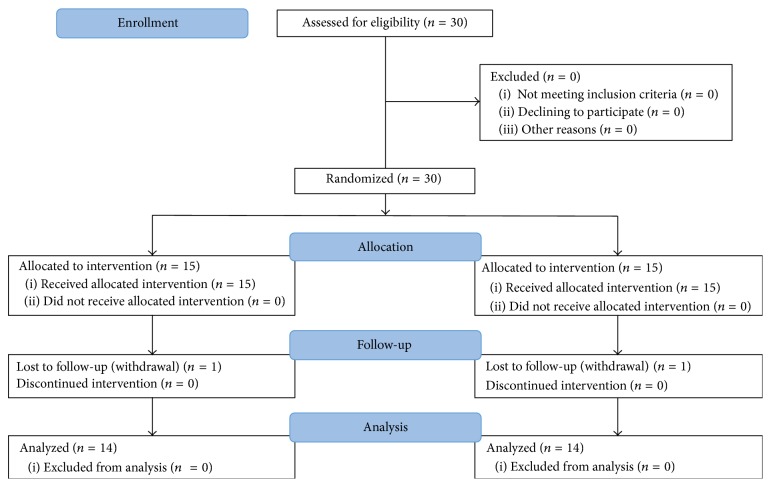
CONSORT flow chart.

Figure 1 shows the CONSORT flow chart. Participants were randomly assigned to an intervention (*n* = 15) or a control group (*n* = 15) using random numbers generated by Microsoft Excel 2010 (Microsoft, Redmond, WA, USA). The randomization was conducted by the study investigators. All participants were blinded to the aims of the study.

**Figure 2:**
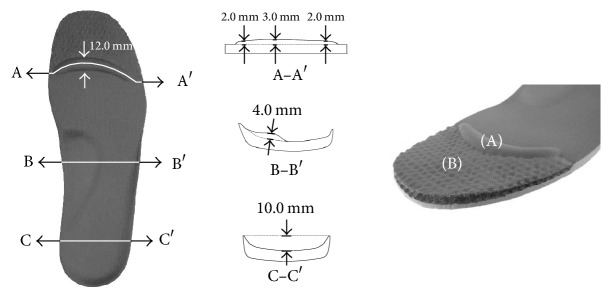
The insoles used in this study consisted of a toe-grip bar (A) and synthetic fiber with high repulsion properties (B).

The intervention group wore shoes with insoles with a toe-grip bar [16]. The main part of the insole is made of synthetic resin foam, while the toe section is made from synthetic fiber with high repulsion properties (known as three-dimensional mesh). Toe-grip bar, which was a convex structure, was placed at the central part of the proximal phalanx from the 1st to 5th toe (Figure 2). The control group wore shoes with general insoles without the toe-grip bar or toe section made from synthetic fiber with high repulsion properties. Otherwise, the structure of the insoles was the same as that for the intervention group. Both groups wore same standard shoes provided by the study investigators, but with different insoles. Both groups wore the shoes for 4 weeks, 5 times per week, 9 hours per day, while at the university. Each day, the study investigators provided the shoes to the participants in the morning and retrieved them 9 hours later, and the participants verbally informed the investigators about the time spent using the insole. All participants fulfilled the total time of intervention described.

### 2.4. Measures

TGS, toe flexibility (TF), total trajectory length (TL), and the envelope area (EA) of the center of pressure were measured, and the functional reach test (FRT) was performed before and immediately after the 4 weeks of intervention.

TGS was measured using the toe-grip dynamometer (T.K.K.3364, Takei Scientific Instruments Co., Ltd., Niigata, Japan); the reliability of the instrument has been previously reported [33]. The subjects were instructed to sit with their trunk in a vertical position, to fold their arms across their chest, to place their hip and knee joints at 90°, and to keep their ankle joints in the neutral position [33–36]. Mean maximum strength was calculated from the maximum strength of two measurements for both sets of toes.

Toe curl ability was used as an index of TF. The foot length and flexed foot length were measured using a scale with the participant in a sitting position. The foot length was defined as the distance from the heel to the tip of the hallux. The flexed foot length was defined as the distance from the heel to the tip of the hallux with maximal toe flexion. Toe curl ability was calculated as the foot length minus the flexed foot length [37, 38]. The mean maximum value was calculated from the maximum value of two measurements for both sets of toes.

Postural sway was measured using a stabilometer (GP-7, Anima Co., Ltd., Tokyo, Japan). The subjects were instructed to stand in the two-leg stance under the following standardized conditions: barefoot, eyes open and looking at a target placed on a wall at eye level 2 m away, and arms at their sides [39]. Data were collected after subjects stood for 5 s to exclude the influence of initial sway. The data were measured for 30 s at a sampling rate of 20 Hz. The mean values of TL [40] and EA [41] of the center of pressure from two measurements were calculated.

FRT was measured using a Functional Reach Measurer (T.K.K. 5802, Takei Scientific Instruments Co., Ltd., Niigata, Japan). The participant stood with the feet approximately shoulder width apart, keeping the forearms pronated, the elbows extended, and the shoulder joints bent at 90° as the starting posture. The participant was asked to reach forward as far as possible while standing [7, 42]. The maximum value from two measurements was used for analysis.

The assessments were conducted by the study investigators. The assessors were not blinded to the participant's group allocations.

### 2.5. Statistical Analysis

The baseline characteristics of the intervention and control groups were compared to check if the two groups were comparable. The Kolmogorov–Smirnov Test was used to test the normality of distributions, and differences between groups were analyzed using Student's *t*-tests for normally distributed variables and the Mann–Whitney *U* test for variables that were not normally distributed. The effect of intervention on outcome measurements was analyzed using mixed 2 × 2 [group (intervention and control groups) × time (pre- and posttest)] analysis of variance. Post hoc Bonferroni testing was used to assess which group or time periods showed significant differences. Statistical analysis was performed with SPSS ver. 23.0 (IBM, Chicago, IL, USA). The level of significance was <5%.

## 3. Results

Two subjects who could not be assessed after training in the intervention and control groups were excluded, leaving 14 subjects in each group. Before the training, there were no significant differences between the groups in age, height, body weight, TGS, TF, FRT, TL, or EA (all *p* > 0.05) (Table 1). Two-way repeated ANOVA showed significant interaction for TGS and TF (*F* = 12.53, *p* < 0.01; *F* = 5.84, *p* < 0.05, resp.). Post hoc Bonferroni comparisons revealed that TGS significantly improved after training in the intervention and control groups (*p* < 0.01, *p* < 0.05, resp.). After training, TGS was significantly higher in the intervention group than in the control group (*p* < 0.01). In the intervention group, TF significantly improved after training (*p* < 0.01). Conversely, no significant interaction was observed among TL, EA, and FRT (Table 2).

## 4. Discussion

TGS and TF significantly improved after training in the intervention group. This suggests that insoles with a toe-grip bar improved TGS and TF in healthy young women.

Our previous study reported that TGS training for 6 weeks using towel gathering improved TGS in healthy young women (after 3 weeks, +2.7 kg; after 6 weeks, +2.9 kg) [18]. In this study, TGS training for 4 weeks using insoles with a toe-grip bar improved TGS in healthy young women (after 4 weeks, +4.2 kg). Our results suggest that the continuous use of the insoles may promote continuous training of the region where improvements in TGS were observed. Therefore, insoles with a toe-grip bar may be an effective tool to improve TGS conveniently.

A significant improvement in TF was observed only in the intervention group. We showed that TF influenced TGS [38]. Another study reported a significant correlation between TGS and TF [35]. A previous study reported that automatic antagonist alpha motor neuron inhibition was evoked by agonist muscle contraction in a phenomenon called reciprocal inhibition [43]. Additionally, antagonist reciprocal inhibition is increased by strength training [44]. Therefore, this study suggests that increased toe-grip movement due to the toe-grip bar facilitates antagonist reciprocal inhibition and improves TF.

Conversely, in the control group, posttraining TGS was significantly improved, which may be due to the influence of improvement and learning effects of measurement methods (pre- and posttest). In this study, significant improvement in TF, which is significantly associated with TGS [35, 38], was only observed in the intervention group. Therefore, improvement and learning effects of the measurement methods may have influenced improvement in TGS in the control group. Otherwise, a placebo effect may have been involved in the TGS improvement shown by the control group. In addition, it is possible that continuous use of the general insole may have promoted TGS. Future studies are needed to exclude factors such as learning effects and the placebo effect.

Training effects for TL, EA, and FRT were not observed in either group; there were no significant differences between the groups. Moreover, mean values were similar to those in previous studies in healthy subjects [16, 45]. Therefore, we suggest that training effects were not observed for the above parameters because this study was on healthy subjects without static or dynamic balance disabilities. Therefore, further studies are needed to investigate the effect of insoles with a toe-grip bar in older adults with disabilities in static and dynamic balance.

Insoles with a toe-grip bar have potential advantages. They have low device costs, can readily be made available, exhibit ease of application, and can be administered by a range of health professionals.

There are some limitations of this study. First, the number of steps during the training period was not measured in this study. Therefore, this study could not exclude the influence of steps during the training period. Further studies are necessary to measure the steps during the training period. Second, it was unclear whether the benefits observed were related to the time spent using the insole. Further studies are needed to investigate whether there is a positive association between these factors, with longer insole use providing greater benefits.

## 5. Conclusions

This study investigated the effects of insoles with a toe-grip bar on toe function and standing balance in healthy young women. TGS and TF significantly improved after training in the intervention group. The results of this study suggest that insoles with a toe-grip bar contribute to improvements in TGS and toe flexibility in healthy young women.

## Figures and Tables

**Table 1:** Characteristics of the intervention (*n* = 14) and control groups (*n* = 14).

Parameter	Intervention		Control		*p* value
	Mean	SD	Mean	SD	
Age (years)	19.86	0.64	20.07	0.70	0.42
Height (cm)	157.36	4.90	156.96	4.59	0.83
Body weight (kg)	51.54	4.41	51.01	5.09	0.78
TGS (kg)	7.58	1.25	7.03	1.31	0.27
TF (cm)	3.31	0.52	3.42	0.80	0.66
FRT (cm)	32.07	7.84	33.50	4.91	0.57
TL (cm)	38.88	6.44	34.57	7.37	0.11
EA (cm^2^)	1.79	0.86	1.81	0.66	0.96

TGS, toe-grip strength; TF, toe flexibility; FRT, functional reach test; TL, total trajectory length; EA, envelope area.

**Table 2:** Comparison of parameters before and after training between the intervention (*n* = 14) and control groups (*n* = 14).

Parameter	Group	Pre		Post		Group × time	
		Mean	SD	Mean	SD	*F*-value	*p* value
TGS (kg)	Intervention	7.58	1.25	11.82	2.51^*∗∗*†^	12.53	0.00
	Control	7.03	1.31	8.29	2.08^*∗*^		

TF (cm)	Intervention	3.31	0.52	3.80	0.62^*∗∗*^	5.84	0.02
	Control	3.42	0.80	3.51	0.78		

FRT (cm)	Intervention	32.07	7.84	34.61	7.90	0.00	1.00
	Control	33.50	4.91	36.04	9.48		

TL (cm)	Intervention	38.88	6.44	37.10	6.03	0.33	0.57
	Control	34.57	7.37	34.02	7.41		

EA (cm^2^)	Intervention	1.79	0.86	1.69	0.69	1.90	0.18
	Control	1.81	0.66	2.04	0.76		

TGS, toe-grip strength; TF, toe flexibility; FRT, functional reach test; TL, total trajectory length; EA, envelope area; ^*∗∗*^*p* < 0.01; significant difference between pre- and posttest; ^*∗*^*p* < 0.05; significant difference between pre- and posttest; ^†^*p* < 0.01; significant difference between intervention and control groups.
